# The Pattern of COVID-19 in Horn of Africa countries, from March-December 2020

**DOI:** 10.4314/ahs.v23i1.13

**Published:** 2023-03

**Authors:** Samson Teweldeberhan Ghebremichael, Rezene Habte Tewolde, Amanuel Kidane Andegiorgish, Guoqing Pan

**Affiliations:** 1 State Key Laboratory of Silkworm Genome Biology, Southwest University, Beibei, Chongqing 400715, China; Chongqing Key Laboratory of Microsporidia Infection and Control, Southwest University, Beibei, Chongqing 400715, China; 2 Department of Biology, Mai Nefhi College of Science, Mai-Nefhi, Eritrea; 3 The State Key Laboratory of Medical Genetics, School of Life Sciences, Central South University, Changsha, Hunan, China; 4 Department of Epidemiology and Biostatistics, School of Public Health, Xi'an Jiaotong University Health Science Center, No 76 West Yanta Road, Xi'an 710061, Shaanxi Province, China

**Keywords:** Coronavirus, COVID-19, pattern, Horn of Africa, fatality rate

## Abstract

**Background:**

Coronavirus-19 (COVID-19) is a novel, highly infectious, and potentially fatal communicable pandemic disease. It has taken longer to reach Africa than the other continents.

**Objective:**

To examine the pattern of COVID-19 in the Horn of Africa countries from March to December 2020.

**Methods:**

A prospective cross-sectional study in which the total number of daily reported cases and deaths were collected from the official website of the World Health Organization (WHO) and Worldometer. Data were standardized based on the total population provided by World Population Prospects 2020 per million. Data sources of WHO reports and governmental reports from March to December 2020 were analyzed in this study. Data extraction was done using the microsoft excel spreadsheet tool, variables relating to the countries were computed in terms of frequencies and percentages.

**Results:**

The findings revealed that as of 31st December 2020, 136,129 (7590 per million) cases were reported from the four countries in the Horn of Africa. The overall case fatality rate (CFR) in the Horn of Africa was 1.14%. The majority of the cases were reported from Djibouti (77.20%), followed by Ethiopia (14.07%), Eritrea (4.87%), and Somalia (3.86%). The highest case fatality rate (0.81%) was from Djibouti, and the lowest (0.01%) was from Eritrea.

**Conclusions:**

The epidemiological pattern of COVID-19 among the Horn of African countries seems to have slow progress, given the prevalence of the new infections remains low, and the death toll seems stable throughout the study periods, except for Djibouti. Hence, the prevention control measures implemented in the countries should be further strengthened and supported. It is recommended that relevant stakeholders responsible for tackling the COVID-19 pandemic should put up measures to curb the spread of the virus in the region and set up a crisis management system to combat future pandemics.

## Introduction

Coronaviruses (CoVs), a subfamily of Orthocoronavirinae in the family of Coronaviridae, Order Nidovirales^1^, has been causing different respiratory diseases such as Middle East Respiratory Syndrome (MERS), severe acute respiratory syndrome (SARS), and the novel coronavirus officially named as COVID-19 (SARS-Cov-2). Coronavirus is one of the major pathogens that primarily target the human respiratory system^2^. Previous outbreaks due to CoVs include the SARS- and the MERS-CoV, characterized as agents of tremendous public health threats^2^. Hereditary arrangement of the COVID-19 indicated over 80% similarity to SARS-CoV^3^,^4^ and 50% to the MERS-CoV, both SARS-CoV and MERS-CoV begun in bats^5^. Similarly, evidence from the phylogenetic analysis also demonstrates that the COVID-19 has a place with the genus betacoronavirus, which contains SARS-CoV, which infects people, bats, and wild creatures^2^.

COVID-19, which was first reported on 31^st^ December 2019 in Wuhan City of Hubei province of China, has spread to the whole world at an alarming rate^6^,^7^. In one month of its initial outbreak, on the 30^th^ of January 2020, the WHO declared that the 2019 novel coronavirus (2019-nCoV) outbreak constitutes a public health emergency of International concern. Since the declaration, many countries have implemented formal preventive measures, including travel restrictions to and from risky areas. The fast spread of the COVID-19 interfered with proper planning by health professionals to mitigate its effect. Adopting emergent measures meant an inadequate focus on health promotion, early detection of infection, disease prevention, and investment in equipping public health professionals with the necessary skills and knowledge to fight the virus. Misinformation and conspiracy theories towards the origin of the pandemic made the globe far away from collaborating on early prevention and mitigation factors. Subsequently, to these global developments, on 11^th^ March 2020, WHO declared COVID-19 as a pandemic^8^.

Although COVID-19 is believed to be a family of the previous outbreaks, the infectivity of COVID-19 is higher than SARS-CoV and MERS-CoV 9. As a result, it is still considered a severe public health threat of this decade. The spill-over effects of the COVID-19 on a diverse aspect of life led the world to experience unimaginable economic, social, and psychological dilemmas. Although many published studies have tried to summarize its impact on different countries, the pandemic still hits on consecutive waves. Further, it aggravates the life of humankind on the globe^1^,^10^–^16^.

Even though COVID-19 is tagged as “the most infectious disease in the last decades”, no antidote has been found to eliminate this canker. The scientific community is still searching for a potent vaccine to safeguard the world from this ravaging pandemic. However, the emergence of new strains of the virus has hampered vaccine development. Presently, the fully embraced practices to break the transmission chain of the virus and ensure high-level community protection at all levels are individual and community-based approaches. At the initial stage of the COVID-19, many countries imposed local and international travel restrictions and various social distancing measures advocated by WHO. COVID-19 was expected to be more dreadful in low-income countries with a fragile economy, inadequate resources and infrastructure for mass screening, limited capacity on accommodation of cases, and lack of the desired therapeutic interventions^16^–^18^.

Nonetheless, COVID-19 took longer to reach Africa's shores than other continents. However, even before the infection arrived at the locale, its extensive impacts were felt^19^. COVID-19 was reported in Africa initially in Egypt on 14^th^ February 2020^25^. The case fatality rate (CFR) of COVID-19 was 2.4% in Africa and 2.2 % globally.

This study is novel in the sense that it is one of the first to examine the pattern of COVID-19 infection in the Horn of Africa and advocate for a crisis-resistant system against future disasters/pandemics. Health officials, governmental agencies, policymakers, NGOs, and significant parties at the helm of authority will benefit from the knowledge of the trajectory of the COVID-19 in the Horn of Africa. Additionally, the study captures the effect of social distancing protocols amid the pandemic. The complexity of adhering to social distancing guidelines is linked to the multiplicity of factors associated with the COVID-19.

Due to contextual factors that fraught countries in the Horn of Africa, the burden of battling the daunting effect of the COVID-19 will be overwhelming if they hit on a similar magnitude to what has been experienced in the middle- and high-income countries. However, recent statistics demonstrate that the effect of the COVID-19 is not as high as professional analysts and the WHO anticipated. Hence, lessons on coverage of crucial prevention measures from countries in the Horn of Africa would aid in budget planning, the precise allocation of resources, and contextualized interventions needed most by these neighbouring countries besides the promoted general health guidelines.

Hence, this study aims to summarize and compare the COVID-19 situation in the four Horn of African countries, namely Djibouti, Eritrea, Ethiopia, and Somalia.

## Methods

### Study design

A prospective cross-sectional study in which the total number of daily reported cases and deaths were collected from the official website of WHO and Worldometer. The study population were all clinically reported confirmed cases of the four Horn of Africa countries (Djibouti, Eritrea, Ethiopia, and Somalia) from March to December 2020. This study examines the patterns of COVID-19 incidences in the four countries of the Horn of Africa Sub-Saharan regions and the extent to which the community has adopted the COVID-19 precaution measures.

### Study settings

The researchers analyzed data from the Horn of African countries. The Horn of Africa is a region of the eastern part of Africa, located closer to the Arabian Peninsula, connecting the Indian Ocean and the Red Sea. It consists of four countries: Djibouti, Eritrea, Ethiopia, and Somalia^26^, ^27^. The people in these countries have interlinked and very close cultural, political and religious connections throughout their long history (Figure I).

**Figure I:**
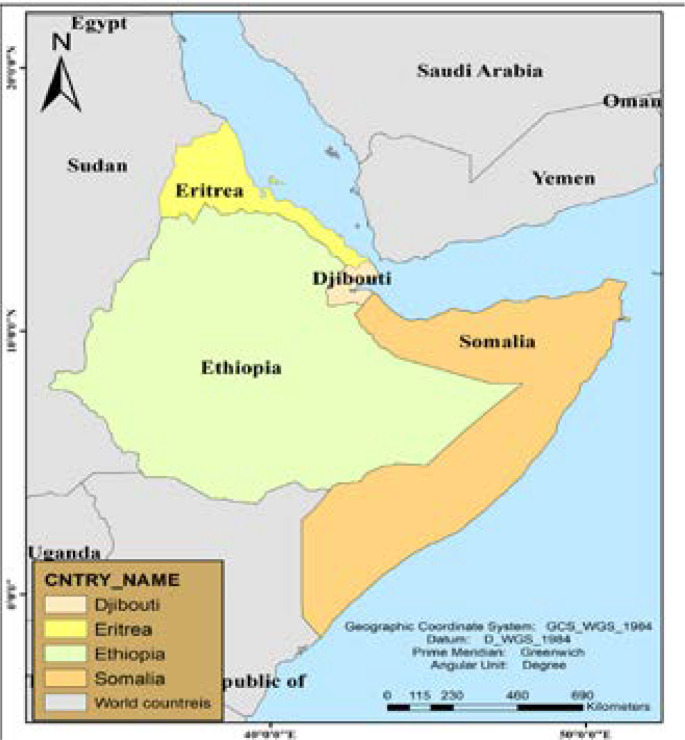
Map of study settings, Horn of Africa 28

### Data collection

These data were obtained from online reports 8, 24 in which daily COVID-19 incidences were collected through an unstructured questionnaire by two independent investigators with core team members. The team met in a small group weekly virtually online via zoom and communicated for any discrepancy on a daily report of the respected country's official information. The researchers made observations from March 2020 to December 2020 on the national number of new cases, deaths, and recovery in each of the selected Horn of Africa region's countries.

### Study population

All daily reported confirmed COVID-19 cases in the studied countries, from residents of the Horn of Africa region, were included in this study.

### Data sources

Daily data recording of two or more trusted sources, mainly the national ministry of health report of each studied country, national media outlets, and WHO report of daily national and global cases, were used. Additionally, information on preventive measures from country sources was included 8, 24, 29–34.

### Statistical analysis

Two independent recorders performed data extraction using the Microsoft Excel Spreadsheet tool. The study's principal investigator carried out rigorous daily quality control monitoring. One of the researchers was assigned to compiling daily records and conducting checks randomly to ensure high-quality data. Descriptive statistics method of data analysis was used to show the distribution of the number of cases and deaths by country. Data were presented using the frequency and percentage of the variables. Standardization of data was based on each country's current total population as provided by the world population prospects 2020^35^ per million.

### Ethical Concerns

This study is exempt from ethical review as it has used publicly available data in which no participant's identification or socio-demographic information was exposed.

## Results

### Description of COVID-19 infection in the Horn of Africa

Table 1 shows the number and percentage of confirmed cases segregated by the studied countries and the total in the Horn Africa region. As of 31^st^ December 2020, the cumulative number of COVID-19 cases detected in the Horn of Africa were 136,129 (7,590 per one million). Country wise distribution or share of these cases was: for Djibouti (77.20%), Eritrea (4.87%), Ethiopia (14.07%), and Somalia (3.86%). The overall case fatality rate (CFR) in the Horn of Africa was 1.14% during the study time. The highest infection and case fatality rates were recorded from Djibouti, which is 5831(5860 per million) and 61(61 per million), respectively. Somalia had the lowest infections, 4714 (293 per million), and Eritrea had the lowest death rate, only three deaths (1 per million) compared to the others. The recovery rate among COVID-19 cases was high in Djibouti (98.2%) and Ethiopia (90.2%), while in Eritrea and Somalia was 51.2% and 76.6%, respectively. The total number of deaths and recovered participants by country is presented in Table 1 below.

**Table 1:** Total number of cases, death, and recovery in the Horn of Africa as the 31^st^ of December 2020

Country	Confirmed cases	Deaths	Recovered	Confirmed cases per 1 M pop (%)	Deaths per 1M pop	Transmission Classification	Recovered per 1M pop
Djibouti	5831	61	5728	5860 (77.2)	61.3	Sporadic cases	5756
Eritrea	1320	3	676	370 (4.87)	0.8	Sporadic cases	189
Ethiopia	124264	1923	112096	1068 (14.07)	16.5	Community transmission	963
Somalia	4714	130	3612	293 (3.86)	8.1	Sporadic cases	224
**Total**	**136129**	**2117**	**122112**	**7,590**	**86.7**		**891**

1M pop= 1,000,000 population

### The monthly incidence of COVID-19 cases and death in the Horn of Africa from March-December 2020

During the study time, in Djibouti alone, a total of 1,068 (1073/1M), 2,256 (2267/1M), and 1328 (1335/1M) new cases were reported in Djibouti as the highest infection in April, May, and June, respectively. However, from July to December 2020, more than 11,600 (100/1M) individuals have been reported in Ethiopia. In May, 29.9% (2267/1M) out of 136,129 (7,590/1M) infected cases in the Horn of Africa were recorded as the highest infection in Djibouti (Table 2, Figure III).

**Table 2:** New Confirmed cases of COID-19 in the Horn of Africa by month in number and standardized per 1,000,000 population

Countries	Mar. N (*)	Apr. N (*)	May N (*)	Jun. N (*)	Jul. N (*)	Aug. N (*)	Sept. N (*)	Oct. N (*)	Nov. N (*)	Dec. N (*)
Djibouti	30 (30.1)	1068 (1073.2)	2256 (2267.1)	1328 (1334.5)	399 (401.0)	306 (307.5)	29 (29.1)	145 (145.7)	116 (116.6)	154 (154.8)
Eritrea	15 (4.2)	24 (6.7)	0 (0.0)	164 (45.9)	76 (21.3)	39 (10.9)	57 (16.0)	88 (24.6)	114 (31.9)	743 (208.1)
Ethiopia	26 (0.2)	105 (0.9)	1041 (8.9)	4674 (40.2)	11684 (100.4)	34601 (297.4)	22453 (193.0)	21585 (185.5)	13905 (119.5)	14190 (121.9)
Somalia	5 (0.3)	596 (37.0)	1375 (85.3)	948 (58.8)	288 (17.9)	98 (6.1)	278 (17.3)	353 (21.9)	510 (31.7)	263 (16.3)
**Total**	**76** **(35)**	**1793** **(1118)**	**4672** **(2361)**	**7114** **(1479)**	**12447** **(541)**	**35044** **(622)**	**22817** **(255)**	**22171** **(378)**	**14645** **(300)**	**15350** **(501)**

N.B (*) Standardized by 1,000,000 population

**Figure III:**
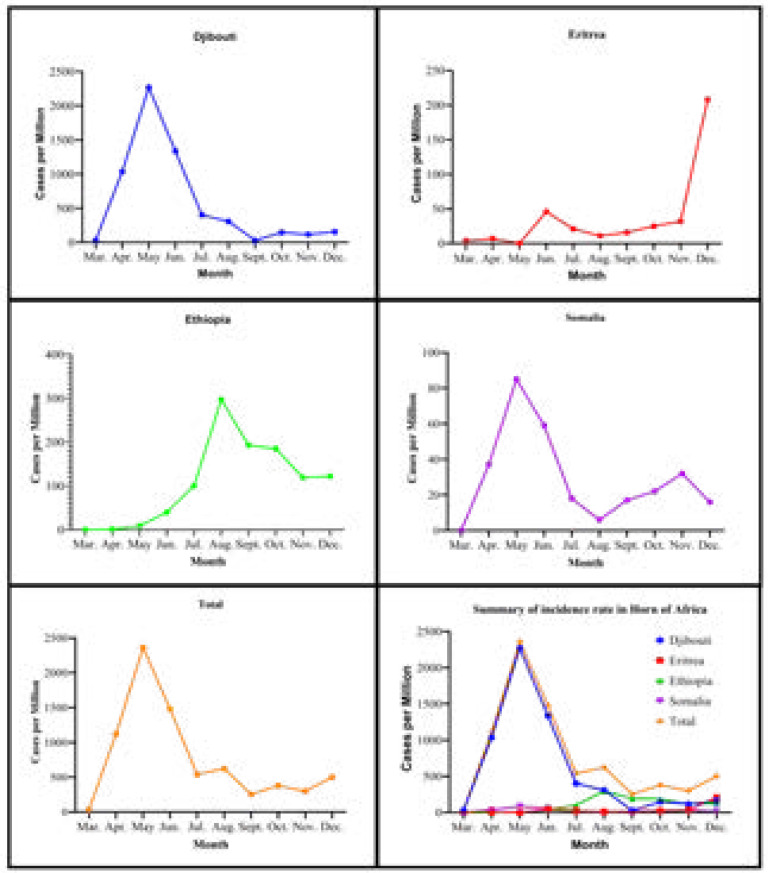
Pattern of new cases of COVID-19 in the Horn of Africa by month from March to December 2020

From Somalia, 50% (2/1M) and 12% (3/1M) out of 33 (4/1M) and 80 (25/1M) death cases were reported in April and May 2020, respectively (Table 3, Figure IV). More than 50% of deaths in the Horn of Africa were recorded from Ethiopia from August to December 2020 (Table 3, Figure IV). In August, the highest proportion of mortality reports was 71.4% (5/1M). Until November 2020, Eritrea was the only country in the Horn of Africa with no deaths related to COVID- 19. However, in December 2020, the country reported three (1/1M) (Table 3, Figure IV).

**Table 3:** Total new deaths per month of COVID-19 in the Horn of Africa in number and standardized per 1,000,000 population

Countries	Mar. N (*)	Apr. N (*)	May N (*)	Jun. N (*)	Jul. N (*)	Aug. N (*)	Sept. N (*)	Oct. N (*)	Nov. N (*)	Dec. N (*)
Djibouti	0 (0)	2 (2.0)	22 (22.1)	30 (30.1)	4 (4.0)	2 (2.0)	1 (1.0)	0 (0.0)	0 (0.0)	0 (0.0)
Eritrea	0 (0.0)	0 (0.0)	0 (0.0)	0 (0.0)	0 (0.0)	0 (0.0)	0 (0.0)	0 (0.0)	0 (0.0)	3 (0.8)
Ethiopia	0 (0.0)	3 (0.0)	8 (0.1)	89 (0.8)	171 (1.5)	535 (4.6)	382 (3.3)	278 (2.4)	237 (2.0)	217 (1.9)
Somalia	0 (0.0)	28 (1.7)	50 (3.1)	12 (0.7)	3 (0.2)	4 (0.2)	2 (0.1)	5 (0.3)	9 (0.6)	17 (1.1)
Total	**0** **(0.0)**	**33** **(4)**	**80** **(25)**	**131** **(32)**	**178** **(6)**	**541** **(7)**	**385** **(4)**	**283** **(3)**	**246** **(3)**	**237** **(4)**

N.B (*) Standardized by 1, 000,000 population

**Figure IV:**
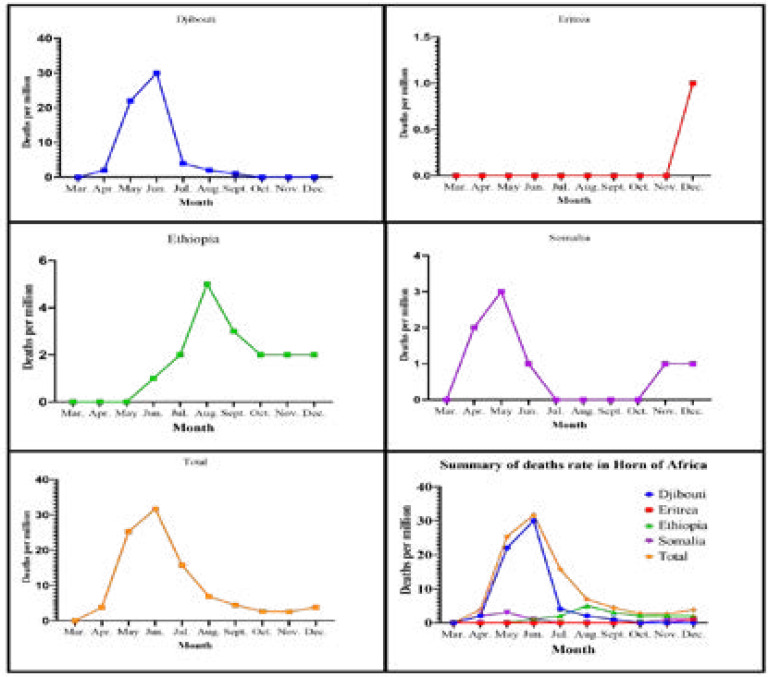
Pattern of COVID-19 deaths in the Horn of Africa by month from March to December 2020

### Intervention measure to mitigate COVID-19 in the Horn of African Countries

The Horn of Africa nations took different preventative measures to prevent the rapid spread of infection to bring the pandemic under control. Throughout the region, strict preventive measures were implemented to slow the spread of the COVID-19. To mention some; complete lockdowns, travel, public gatherings ban (including sports, religious, social, and other occasions), closure of; schools, companies, offices, systematic quarantines, and increased testing capacity 36.

Djibouti's government received 5 million U.S dollars as an emergency fund approved from the World Bank on the 2^nd^ of April 2020^29^. Moreover, WHO provided personal protective equipment as a preventative measure to Djibouti. In contrast, Somalia assigned 5 million U.S. dollars to fight the pandemic^23^. An intensive campaign was given to the community using different mass media and community levels in Eritrea. The Ministry of Health announced quarantine for visitors to Eritrea from the epicentre countries beginning from 1^th^ March 2020 37.

Moreover, all schools were closed, social distancing in all religious places was also applied following the international standard protocols. Except for those with an approved license (permission), all public transport at regional levels and cities were suspended. Eritrean diaspora communities donated a huge amount of money to support the government in fighting the pandemic 38 and ensuring the well-being of nationals inside the country. Besides, many house owners' citizens living inside and abroad are offered at least two months to one-year free rental payment to their tenants.

In Djibouti, the government announced a national lockdown on 23^rd^ March and extended it to the 8^th^ of May^39^. In line with this, all schools and worship places have been closed since 19^th^ and 22^nd^ March, respectively. Ethiopia's government has quickly taken various measures to prevent the spread and control the pandemic^34^. For instance, the authorities have closed all boundaries, shut down schools, universities, and colleges, requested the shuttering of nightclubs and amusement outlets, reported social distancing measures, called on retired healthcare professionals for support, and prepared clinical faculty for National Health Service. Moreover, all individuals entering Ethiopia from abroad were subjected to the mandatory 14-day isolation at assigned hotels at their own cost. On the 8^th^ of April 2020, Ethiopia banned inter-regional public transport and public gatherings except for the Djibouti border to transport commercial goods^40^.

## Discussion

The emergent nature of the COVID-19 has stimulated huge community tensions in both developed and developing countries. The numbers of COVID-19 cases identified in the Horn of Africa up to the 31^st^ of December 2020 were 136,129, accounted for 0.16% of the total 83,068,034 cases reported worldwide. Most of the cases, 5860/1M and death 61/1M in the Horn of Africa, were from Djibouti. The overall incidence in the Horn of Africa was low compared to other African countries in the same time interval. For comparison purpose only, such as South Africa (cases=1,059,161, death=28,921/1.8%), Morocco (cases=439,193, death=7,388/1.7%), Tunisia (cases=139,140, death=4,676/3.4%) and Egypt (cases= 138,062, death=7,631/5.5%)^24^.

Ever since the first case was detected, each government of the Horn of African countries immediately took various sweeping measures to reduce the impacts of the COVID-19. The measures included but are not limited to; schools' closure, travel restrictions, ban on public gathering, nationwide lockdown, mandatory quarantine; for any entering the country, those who had direct contact with a confirmed case, and communities or buildings where a case was detected^39^,^41^. Likewise, the other strategies were suspended public transport and imposed travel restrictions and task force formation responses to COVID-19^38^. Horn of Africa's layered contact-tracing procedure has additionally demonstrated basic quality in detecting the infection. International incoming flights were suspended in Djibouti and Somalia on 18^th^ March^42^,^43^, Eritrea on 25^th^ March^37^, and Ethiopia on 20^th^ and 29^th^ March 2020 to more than 30 and 80 countries affected with the coronavirus, respectively^44^,^45^.

As of 18^th^ July, WHO mentioned Ethiopia among the top ten African nations accounting for 88 % of all COVID-19 cases in the African region^46^. In the country, new infections became less pronounced in March and April after the first appearance of COVID-19 in the Horn of Africa in March. In May and August, in Djibouti and Ethiopia, the highest reported cases were 2267/1M (Figure III) and death 5/1M (Figure IV), respectively. However, the incidence and deaths of COVID-19 have decreased from September to December in Ethiopia. In Somalia, the case fatality rate increased from July (1.1%) to December (6.9%) (Figure IV). Djibouti's socio-economic situation forced the Djiboutian government's hand to lift implemented lockdown measures, resulting in the country's increased infection rate^47^. The high number of infections in Ethiopia could be due to the only partial and not complete lockdown measure implemented, the highly dense population in Addis Ababa, the capital^48^, and delayed suspension of incoming international flights^49^. These might have contributed to the fast spread in local transmission of the diseases to other regions of the country^50^.

Similarly, Mohammed and colleagues' study showed that most of the cases reported were from Addis Ababa^51^. It is worth mentioning that this was similar to the prediction of COVID-19 in East Africa countries by Takele 34. Besides, the knowledge, attitude, and practice towards the prevention measures' practice were not encouraging to tackle and reduce the influence of COVID-19 48, 50. All these might have contributed to the high incidence in Ethiopia compared to remaining Horn of Africa countries.

Overall, the Horn of Africa's death rate was low compared to the developed countries (those nations in Europe, North America, Asia, South America, and so forth). Thus, it could probably be due to the average population's young ages^52^. Previous literature showed that Africa's population is only 4% older than 65, which is much lower when compared with 37% in Eastern and South-Eastern Asia and 29% in Europe and Northern America^53^. The young age group has less susceptibility and better response whenever contracted with the diseases.

Djibouti was listed as one of the high recorded coronavirus infections in Africa on 24th April, and with 1068 (1073/1M) confirmed new cases^54^. A study by Gayawan and his colleagues reported similar Spatio-temporal epidemic dynamics of COVID-19^55^. Similarly, the incidence was high from April to June in Somalia and Djibouti. However, the incidence decreased significantly in Somalia and Djibouti from July to September. Nevertheless, the number of new cases increased from October to December, consistent with the study conducted by Takele^34^. Since June 2020, the confirmed COVID-19 cases in Ethiopia have increased significantly and made the country to be listed as one of the tenth and hundred highly affected countries in the Horn of Africa, and the world, respectively^24^.

Eritrea has the lowest death figures 1/1M compared to the other Horn of Africa. However, the incidence rate was higher than Burundi (Case= 818, death= 2), Tanzania (Case= 509, death= 21) and Mauritius (Case= 527, death= 10)^56^ in the same time frame. From June 2020 onward, the lockdown measures eased nationwide, and the restriction only remained to local transportation and international flights^57^ that could be a reason for the slow increase of COVID-19 incidence in Eritrea. Another study conducted in Italy revealed large-scale lockdown implementation measures reduced the number of COVID-19 infections^58^.

Somalia recorded high mortality rates in April (50%) and May (12%). A study by Ahmed and his colleagues stated low adherence to COVID-19 preventative measures 41, which probably led to a high number of infections and deaths. In contrast, Eritrea remained with zero death cases until its first report of deaths on the last week of December 2020 with a total of three (1/1M) deaths^37^, while, in Ethiopia, Somalia, and Djibouti, their first patients' deaths from COVID-19 recorded in early April^59^,^60^.

As cases worsen, a rapid response measure is essential to be put forward in halting the spread of the pandemic. Thus, subduing the COVID-19 was given a high priority for many countries. In response, Eritrea applied stricter measures that attained a substantial outcome in the rate of infection and death cases 61, 62. In addition, lockdown measures and early implementation were related to positive results 63. Other reasons for the reduced cases and deaths in the Horn of Africa, particularly in Eritrea, were the demographics, low volume of global air traffic, and a moderately young population that played a positive part in slowing down the pandemic 62. In general, as many studies have reported diseases associated with severe illness and death from COVID-19, regularly connected with older populations, such as for overweight, hypertension, cardiovascular diseases, chronic lung infection, malignancy, and diabetes, are less predominant in the region^52^,^62^,^64^.

Although burgeoning literature refers to Horn of Africa countries as economically fragile states^16^–^18^, they were adequately prepared for the pandemic as it took a long time to reach the region compared to other continents. This preparedness could be one reason for the low cases in the Horn of Africa. This observation is similar to what has been reported in West Africa^65^. To our knowledge, there was no delay concerning reporting, underreporting, or misdiagnosis in the Horn of Africa countries.

## Conclusions

The Horn of Africa took several steps to detect, manage and control COVID-19. The epidemiological pattern of COVID-19 among the Horn of African countries seems to have slow progress, given the prevalence of the new infections remains low, and the death toll seems stable throughout the study periods, except for Djibouti. Hence, the pandemic needs timely consideration; and prevention and control measures to curb and mitigate the transmission of the infection and minimize death rate need to be further strengthened. In general, the Horn of Africa countries need to continue in harmony and collaboration to work together in solidarity to safeguard their population from the pandemic.

To the best of our knowledge, this is the first study to provide a trend on the COVID-19 using data from the four Horn of Africa countries. Nevertheless, this is not without a limitation. The limitation of our study is that the age and gender data of the Horn of Africa countries in the official website of the WHO and Worldometer were not available. Hence, the researchers could not compare based on age and gender. Also, since this study focused on the Horn of Africa countries, the situation in other parts of Africa wasn't reported. Comparisons were made regarding the previously mentioned countries in the Horn of Africa. Future researchers are encouraged to conduct a similar study that compares data based on demographic variables such as age and gender and captures statistical data on the pandemic situation in other African countries.

## Figures and Tables

**Figure II:**
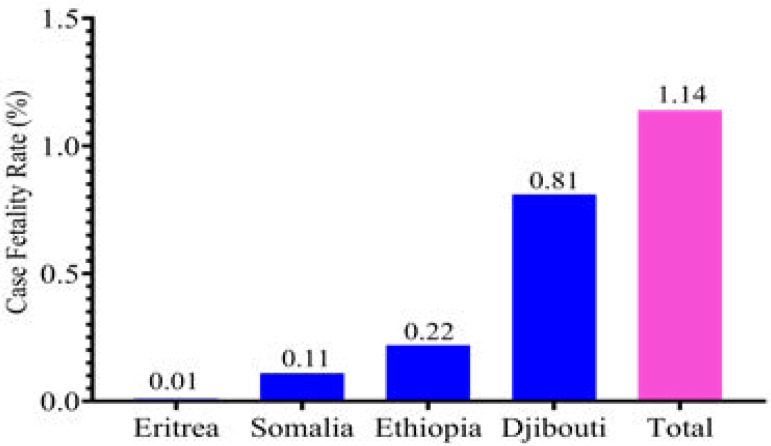
Overall COVID-19 case fatality by the studied country and the Horn of Africa, up to the 31^st^ of December 2020
